# Bioengineering Pancreatic Organoids and iPSC-Derived β-Cells for Diabetes: Materials, Devices, and Translational Challenges

**DOI:** 10.3390/bioengineering13040478

**Published:** 2026-04-18

**Authors:** Abdullah Jabri, Mohamed Alsharif, Bader Taftafa, Tasnim Abbad, Dania Sibai, Abdulaziz Mhannayeh, Abdulrahman Elsalti, Islam M. Saadeldin, Jahan Salma, Tanveer Ahmad Mir, Ahmed Yaqinuddin

**Affiliations:** 1 College of Medicine, Alfaisal University, Riyadh 11533, Saudi Arabiamoalsharif@alfaisal.edu (M.A.); betaftafa@alfaisal.edu (B.T.); tabbad@alfaisal.edu (T.A.); dalsebaai@alfaisal.edu (D.S.); amohanaya@alfaisal.edu (A.M.); imohamed@kfshrc.edu.sa (I.M.S.); tmir@kfshrc.edu.sa (T.A.M.); 2 Department of Internal Medicine, Acıbadem MAA University, Istanbul 34752, Turkey; abdulrahman.el@std.medipol.edu.tr; 3 Research Laboratories, King Faisal Specialist Hospital and Research Centre, Riyadh 11211, Saudi Arabia; 4 Laboratory of Tissue/Organ Bioengineering & BioMEMS, Organ Transplant Centre of Excellence (TR&I-Dpt), King Faisal Specialist Hospital & Research Centre, Riyadh 11211, Saudi Arabia; jsalma@kfshrc.edu.sa

**Keywords:** pancreas, organoids, pluripotent stem cells, diabetes mellitus

## Abstract

Diabetes mellitus is primarily caused by the loss or malfunction of insulin-producing β-cells, and although current therapies improve glycemic control, they do not restore physiologic insulin secretion. Advances in stem cell biology and organoid engineering have led to the development of pancreatic organoids and induced pluripotent stem cell (iPSC)-derived β-cells as promising platforms for disease modeling, drug testing, and regenerative medicine. Pancreatic organoids generated from ductal, acinar, or progenitor populations can recapitulate key anatomical and functional features of native pancreatic tissue, enabling studies of development, injury, and regeneration. In parallel, improvements in iPSC differentiation protocols have produced β-like cells capable of insulin secretion in response to glucose, although achieving full functional maturity remains a challenge. Bioengineering strategies, including biomaterial scaffolds, microfluidic platforms, endothelial co-culture systems, three-dimensional bioprinting, and CRISPR-based genome editing, have enhanced the stability, vascular compatibility, and functional performance of both organoid and iPSC-derived systems. Despite these advances, variability in differentiation efficiency, limited β-cell maturity, and poor long-term survival continue to hinder clinical translation. Together, pancreatic organoids and iPSC-derived β-cells represent complementary platforms that advance fundamental research and support the development of β-cell replacement therapies, with ongoing integration of bioengineering approaches expected to accelerate progress toward reproducible, scalable, and clinically relevant β-cell regeneration.

## 1. Introduction

Diabetes mellitus (DM) remains a premier global health challenge, defined by the progressive failure or autoimmune destruction of pancreatic β-cells. While exogenous insulin therapy and cadaveric islet transplantation have provided life-saving interventions, they are perpetually hampered by non-physiological glucose kinetics, donor shortages, and the requirement for chronic immunosuppression [1,2]. Consequently, the field has pivoted toward de novo generation of functional β-cells using induced pluripotent stem cell (iPSC) technology and three-dimensional (3D) pancreatic organoids [3,4].

They could be applied in research, drug discovery, and regenerative medicine. iPSCs serve as a source of reprogrammed cells for examining pancreatic development and diseases [5]. In addition, organoids offer a 3D model of pancreatic tissue that mimics its structure, function, and interactions between cells. These methods represent a major step forward in addressing the current challenges in DM treatment.

While existing literature has extensively reviewed the developmental biology of β-cell differentiation or the general potential of stem cell therapies, this review specifically addresses the bioengineering-translational gap. We provide a systematic comparison between iPSC-derived platforms and adult tissue-derived organoids, focusing on how engineering interventions are being deployed to overcome clinical obstacles. By mapping these engineering strategies to specific translational hurdles, this review positions these technologies not just as models of disease, but as viable candidates for the next generation of cell-replacement therapies.

## 2. Pancreatic β-Cell Organoids

**Figure 1:**
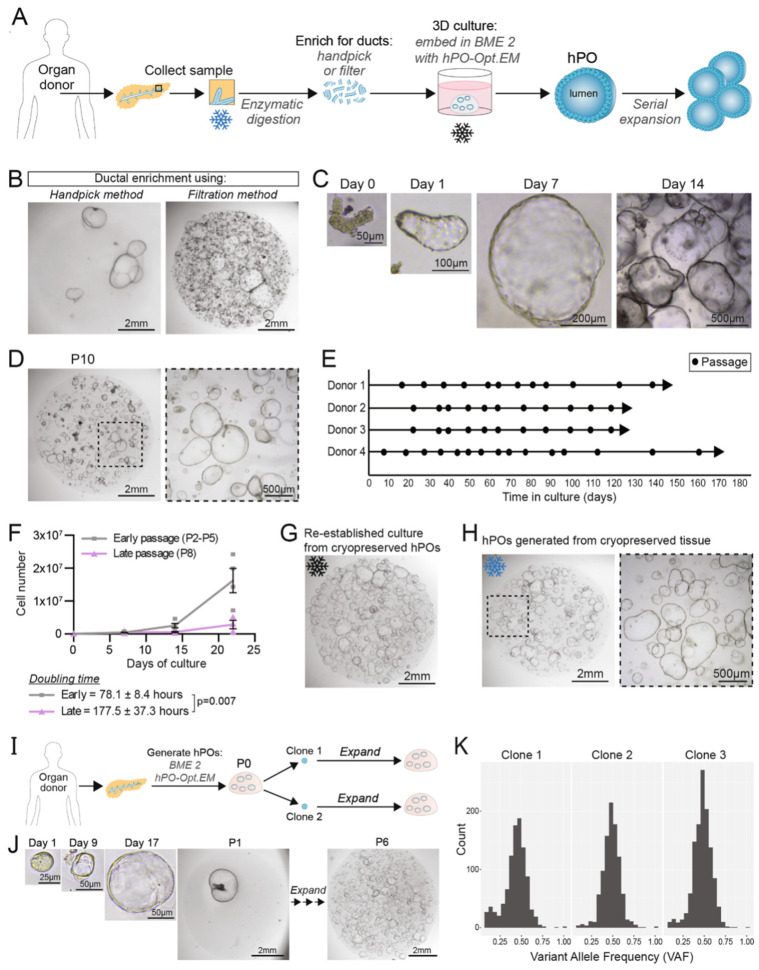
(**A**) Schematic representation of human pancreatic organoids generation and expansion. (**B**) Comparison of hPOs cultures following ductal enrichment by handpicking (left) or filtration (right) methods (*n* = 5). (**C**) Brightfield images of hPOs at different time points during growth. (**D**) Representative brightfield images after long-term culture of hPO (passage 10). (**E**) Culture and expansion of hPOs over several months (*n* = 4 independent donors; circles represent passages). (**F**) The graph shows the growth of independent donors during early and late passages (early gray *n* = 4; late passage, purple, *n* = 3). (**G**) Representative image of hPOs derived from cryopreserved fragments (*n* = 9 independent donors). (**H**) Representative images show hPOs generated from cryopreserved (3 weeks) primary human pancreatic tissue (*n* = 3). (**I**) Workflow depicts clonal cultures generated from hPO cells derived from organoids (P0). (**J**) Representative images of hPO cells and clonal organoids (*n* = 5 independent donors). (**K**) The variant allele frequencies of single-nucleotide variants analyzed using genome sequencing technology. Figure 1—Reproduced from Georgakopoulos et al., 2020, with copyright permission under the terms of the CC-BY 4.0 license [4].

Organoids are 3D tissues grown in vitro from adult stem cells (ASCs) or pluripotent stem cells (PSCs), closely mimicking the structural features and physiological functions of their respective organs in the body [6]. Organoids have been successfully developed for a variety of tissues, including the endometrium, pancreas, heart, and liver [7,8,9]. Organoid models recapitulate the key architecture and functions of their source organs, enabling more faithful studies of development, disease, and drug response across the reproductive, cardiovascular, and hepatic systems [10]. Pancreatic organoids derived from normal pancreatic tissue or from pancreatic cancers provide a practical bridge between in vitro assays and in vivo studies, allowing more precise interrogation of pancreatic biology and pathology [11]. Figure 1 outlines the derivation workflow and demonstrates the long-term expansion capacity of human pancreatic organoids.

### 2.1. Cell Sources for Generating Pancreatic β-Cells Organoids (Table 1)

#### 2.1.1. Adult Stem Cell-Based Formation

Recent developments have improved the derivation of pancreatic β-cell organoids from embryonic and adult stem cells in addition to previously established protocols. Because of their pluripotency and ability to self-renew, embryonic stem cells (ESCs) continue to be essential for the development of insulin-producing cells [12,13]. The creation of functional β-like cells that react dynamically to glucose stimulation has been made possible by stepwise differentiation protocols that mimic embryonic pancreatic development. Key signaling molecules that direct ESCs through definitive endoderm, pancreatic progenitor, and endocrine stages, which are identified by the expression of PDX1, NKX6.1, and insulin, are usually sequentially exposed in these protocols [14]. Furthermore, it has been demonstrated that using 3D culture settings and clustering techniques improves β-cell maturation and insulin secretory capacity.

Organoids grown from ASCs in pancreatic duct tissue can be guided toward endocrine-like phenotypes. When cultured in extracellular matrix gels such as Matrigel^®^ and supplemented with growth cues like EGF, Noggin, and R-spondin, they form stable organoids that maintain genetic integrity [4]. These organoids retain progenitor characteristics and, under specific conditions, exhibit endocrine differentiation potential, making them suitable for disease modeling and autologous transplantation approaches [15].

#### 2.1.2. Tissue-Derived Progenitor-Based Organoid Formation

An interesting observation in recent studies indicates the existence of potential pancreatic progenitor cells within acinar, ductal, and islet compartments of the pancreas [16,17,18]. Notably, these various cell populations have demonstrated the ability to promote β-cell regeneration both under typical circumstances and in reaction to various stressors [19,20,21]. More recent work has identified marker-defined progenitor subsets with enhanced regenerative and expansion potential. In particular, a resident protein C receptor–positive (Procr^+^) population has been shown to support long-term expansion of pancreatic islet organoids while maintaining endocrine competence in vitro, providing a defined tissue-resident source for scalable organoid lines [22]. Incorporating Procr^+^ selection into derivation workflows can improve clonogenicity and stability, while enabling standardized propagation for downstream studies. Because they retain the pancreas’s own regenerative programs, pancreatic tissue–derived cells are a practical starting point for engineering islet organoids. Unlike human pluripotent stem cells (hPSCs) or generic ASCs, they share a developmental origin and epigenetic memory with islet cells, which can support more efficient differentiation into insulin-producing organoids [23]. Moreover, recent research has shown that these cells can differentiate into insulin-producing β-cells in both in vivo and in vitro settings [21,24].

### 2.2. Methods for Generating Pancreatic β-Cells Organoids

#### 2.2.1. Traditional Self Aggregation Method

The process by which dissociated cells spontaneously come together to form tissue-like structures is called cellular self-aggregation, or self-organization, and it is commonly seen in developmental biology [25]. Soft extracellular matrix (ECM) substrates, accessory cells, and low-adherence culture surfaces are some of the factors that can improve this process [26]. Low-attachment culture plates, for example, can enhance the spontaneous aggregation and formation of 3D islet-like organoids from endocrine cells, β-cells, or pancreatic progenitor cells derived from hPSCs [27,28]. This is further supported by the fact that human embryonic stem cells (hESCs) can form embryoid bodies in suspension cultures using spinning flask systems, which then go through a staged differentiation process to become islet organoids [29,30]. As evidenced by improved insulin secretion and vascularization in transplanted models, other cell types, such as mesenchymal stem cells, not only assist in establishing self-aggregation but also improve in vivo functionality [31]. Furthermore, through facilitating the development of homogeneous pancreatic spheroids with more robust islet phenotypes, soft biomaterials such as Amikagel hydrogel have outperformed Matrigel. Although their precise mechanisms are still being studied, novel materials like recombinant silk matrices also show promise in promoting organoid assembly [32]. However, even though conventional self-aggregation techniques can produce functionally complex organoids, the lack of external patterning cues frequently leads to heterogeneous populations, with varied aggregate size and cell number being crucial factors in determining the results of differentiation [33].

#### 2.2.2. Controlled Self Aggregation Method

A key factor in the successful production of human islet organoids is the size of the cell clusters. Whether in vitro or after implantation, larger islets are more likely to necrotize and have lower cell viability than smaller ones [34]. Therefore, instead of depending on conventional, unregulated methods, a controlled self-aggregation method is required [33]. Alternative methods, such as hanging drop culture and micro-contact printing, have been developed to allow for more controlled cell self-aggregation and produce uniformly sized organoids. The hanging drop technique, which uses gravity to induce cell clustering, is widely utilized for producing embryoid bodies and requires no contact with artificial scaffolds or surfaces. Montanari et al. produced functional islet organoids by mixing dissociated human islet cells with mesenchymal cells in suspended droplets, demonstrating insulin secretion in vivo [35,36]. This approach enables for control over organoid size by manipulating cell numbers, droplet volume, and incubation time. Despite its precision, it is labor-intensive and not ideal for large-scale production. Mendelsohn et al. created a micro-contact printing process in which the cell-adhesive protein laminin is covalently printed on glass coverslips [37]. This approach allows for fine control over the shape and size of cell clusters, while high-speed printing speeds up the generation of β-cell aggregates. However, these clusters only have two to three cell layers and cannot be separated from the substrates, making them unsuitable for pancreatic islet models, biological testing, or transplantation [38].

### 2.3. Key Advancements in Organoid Technology

#### 2.3.1. Microfluidic Platforms: Enhancing Physiological Relevance

**Figure 2:**
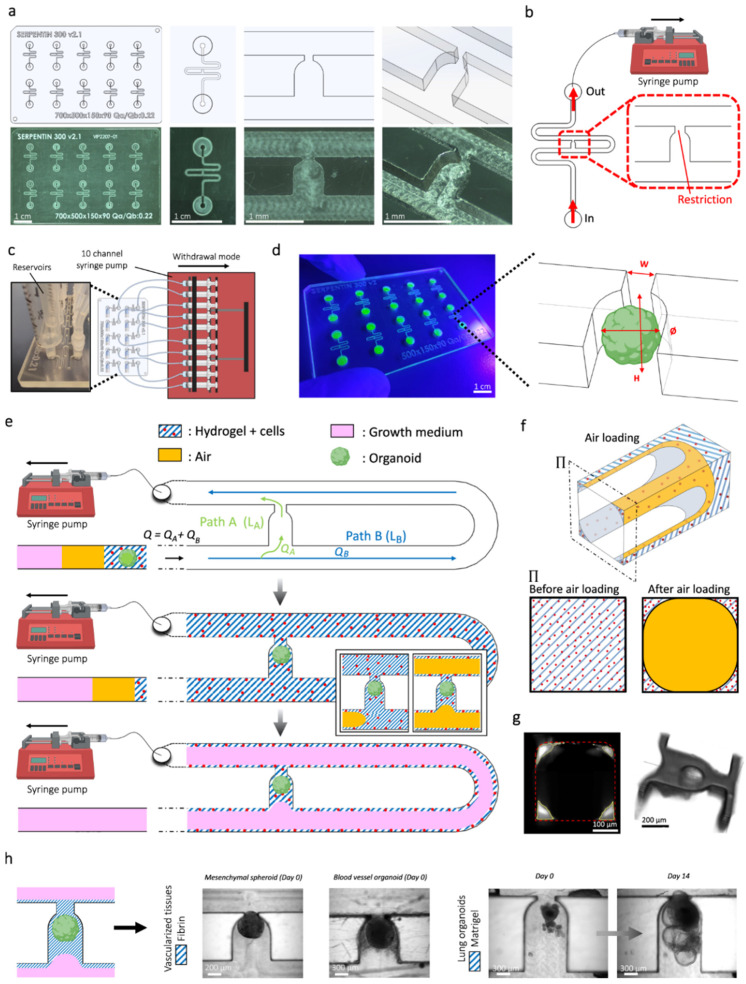
Microfluidics chip-based microphysiological system design and overview of organoid culture and cell configurations. (**a**) Computer-aided design (top) and photographs (bottom) of the microfluidic chip showing multiple microchannels. (**b**) Top view of the device connected to a syringe pump. (**c**) Schematic diagram and photograph of the microchannels. (**d**) Photograph of the microfluidic chip device and schematic view of the U-cup-shaped area functioning as a trap. (**e**) Schematic diagram showing an overview of the loading process of the hydrogel and perfusion of the hydrogel loaded with organoid and HUVEC cells. (**f**) Schematic depiction of the microchannel displaying the air loading process and deposition of the hydrogel. (**g**) Cross-sectional view (left) of the microfluidic channel displaying the deposition of the hydrogel in the trap and in the channel’s corners, and 3D rendering (right). (**h**) Representative micrographs of vascular spheroid/organoid embedded in fibrin-based hydrogel (left), and hiPSC-derived lung organoids maintained in Matrigel-based hydrogel, indicating the trapping and growth over an extended period of time—two weeks on-chip (right). Figure 2—Reproduced from Quintard et al., 2024, with copyright permission under the terms of the CC-BY 4.0 license [41].

Because they provide dynamic and physiologically relevant environments, microfluidic “organ-on-a-chip” systems have become revolutionary tools in islet organoid culture. By closely simulating in vivo conditions, these platforms enable precise control over waste removal, mechanical stimuli, and the delivery of nutrients and oxygen, as illustrated in Figure 2 [39]. For example, Wang et al. showed that microfluidic devices could facilitate the long-term culture and functional evaluation of islet organoids, offering a promising path for diabetes research and treatment [40]. Vascular networks have recently been incorporated into microfluidic systems, which has improved the maturation and functionality of islet organoids even more. The creation of a microfluidic platform that effectively vascularized a variety of biological tissues, including pancreatic islet spheroids, is a noteworthy example. This enhanced organoid growth and metabolic function [41].

#### 2.3.2. 3D Bioprinting

The production of islet organoids has been revolutionized by 3D bioprinting, which makes it possible to precisely organize different cell types within biomimetic scaffolds [42]. Researchers have successfully produced islet-like structures that preserve cell viability and insulin secretion using bioinks made of ECM components and pancreatic cells. For instance, a study demonstrated how 3D bioprinting can be used to create islet-specific niches, which will help stem cell-derived β-cells mature [43]. In the context of islet engineering, polylactic acid (PLA) and fibrin hydrogels have been utilized to encapsulate hPSC-derived β-cell clusters using 3D printing techniques. When transplanted into mice, these 3D-printed structures containing β-cells remained functional for up to 12 weeks, could be safely retrieved, and preserved their structural stability throughout the implantation period [44]. Primary pancreatic islets have been directly bioprinted using tissue-specific bioinks [45]. A hyaluronic-acid-methacrylate/pancreatic decellularized ECM (HAMA/pECM) hybrid supported 3D-printed islet organoids that preserved islet morphology via Rac1/ROCK/MLCK signaling, improved glucose-stimulated insulin release in vitro, promoted vascular ingrowth, and maintained normoglycemia for ~90 days after implantation in diabetic mice [46]. In a complementary in vivo study, porcine islets bioprinted into “bionic scaffold petals” and transplanted subcutaneously in NOD-SCID mice lowered fasting glucose and showed neovascularization [47]. It also supports the maturation of insulin-producing cells.

#### 2.3.3. Co-Culture Systems

Co-culture systems have been developed to improve organoid maturation through the interaction of pancreatic β-cells with other cell types, such as immune cells, mesenchymal stem cells, or endothelial cells. The intricate cellular interactions found in the natural islet microenvironment are what these systems seek to mimic [48,49]. One promising method for producing functional pancreatic pseudo-tissues for both in vitro and in vivo applications is co-culturing β-cells with endothelial cells. In a recent study, endothelial layers encircled islet-like clusters formed when MIN6 (β-like) and MS1 (endothelial) cells were co-cultured within micro-patterned collagen sheets [50]. The significance of controlled microenvironments for mimicking native pancreatic architecture was demonstrated by this structured 3D environment, which promoted vascular-like organization and increased insulin secretion. Moreover, co-culture with liver organoids in microfluidic systems has demonstrated enhanced glucose metabolism, highlighting the importance of inter-organ communication in maintaining glucose homeostasis [51].

### 2.4. Functional Assessment of Pancreatic β-Cells

**Figure 3:**
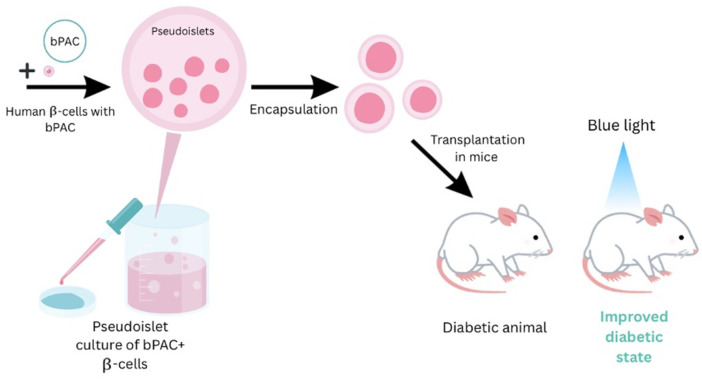
This figure depicts an optogenetic strategy to enhance insulin secretion in human stem cell–derived β-cells. By expressing the light-sensitive enzyme bPAC, these engineered cells form pseudoislets, are encapsulated, and transplanted into diabetic mice. Blue light activates insulin release in vivo, improving glycemic control without drugs. The image is adapted from Chen, Z. et al. (2024), with a copyright permission [52].

Recent discoveries in DM research have considerably expanded our understanding of glucose-stimulated insulin secretion (GSIS), β-cell electrophysiology, and engineered pancreatic tissues’ in vivo engraftment capability. GSIS is a key function of pancreatic β-cells that produce insulin in response to high blood glucose levels. Several studies aim to boost GSIS in stem cell-derived β-cells to mimic the functionality of native pancreatic tissue. One particularly intriguing option is the use of optogenetics to control insulin secretion [52]. Chen, Z. et al. inserted light-sensitive ion channels into human stem cell-derived β-cells, allowing for precise regulation of electrophysiological properties using blue light, as illustrated in Figure 3. This stimulation increased membrane depolarization, calcium influx, and potassium channel activity, resulting in a substantial rise in GSIS. The modified β-cells functioned well in vitro and sustained glycemic control when transplanted into diabetic mouse models, indicating therapeutic viability and functional engraftment [53]. These findings highlight the potential of optogenetics as a non-invasive, temporally regulated approach for fine-tuning insulin production without pharmaceutical intervention.

In addition to optogenetic techniques, additional research has demonstrated the profound heterogeneity in GSIS among individual islets. Using a microfluidic device for real-time single-islet study, researchers revealed significant diversity in insulin secretion dynamics among isolated islets, with some responding strongly to glucose and others remaining largely inactive [54]. This variability emphasizes the need to select high-functioning islets for transplantation, as well as the possibility that not all islets in a single donor contribute equally to glucose management. By improving our understanding of these functional distinctions, such platforms have the potential to greatly increase the efficacy of islet-based cell treatments. GSIS is connected to β-cell electrophysiology, specifically calcium flow and potassium channel activation [55]. Glucose metabolism in β-cells produces ATP, which blocks ATP-sensitive potassium (K_ATP) channels. This causes membrane depolarization and opens voltage-gated calcium channels. The ensuing calcium influx is the major driver of insulin granule exocytosis. The functional heterogeneity of β-cells can be explained by variations in the expression or sensitivity of these channels, making them prospective targets for therapeutic augmentation.

To gain a more comprehensive understanding of calcium dynamics, a study comparing experimental data and computational models was conducted, highlighting the role of intracellular calcium stores such as the endoplasmic reticulum, alongside extracellular calcium influx through voltage-dependent channels [56]. This integrative approach gave new insights into how cellular signaling cascades are organized during GSIS. It indicated that β-cell populations display varied calcium signaling patterns, which can alter insulin secretion efficiency. These findings are critical for designing β-cell replacements and organoids to ensure consistent and strong insulin release. In vivo engraftment studies in diabetic mouse models are crucial for evaluating the functionality and durability of bioengineered β-cells and organoids, in addition to in vitro findings. A comprehensive evaluation of islet transplantation methodology stressed the importance of consistency in DM induction regimens, islet isolation techniques, and post-transplantation monitoring to achieve accurate results in preclinical models [57].

One novel technique for improving in vivo engraftment was the creation of insulin-producing organoids by co-culturing dissociated islet cells with human amniotic epithelial cells (hAECs) [58]. These hybrid organoids showed better structural integrity, vascularization, and functional performance, restoring normoglycemia in type 1 diabetic (T1D) mice models. The integration of hAECs not only improved cell survival but also helped to create functional microenvironments conducive to insulin secretion. Another interesting strategy used hiPSCs co-cultured with human endothelial cells to create pancreatic organoids in a chemically defined environment [59]. These organoids showed mature β-cell features, including expression of essential markers (PDX-1, GLUT-2), responsiveness to glucose, and suitable electrophysiological qualities, such as controlled calcium and potassium channel activity. When transplanted into diabetic mice, the organoids quickly restored blood glucose levels, indicating both successful engraftment and functional integration.

**Figure 4:**
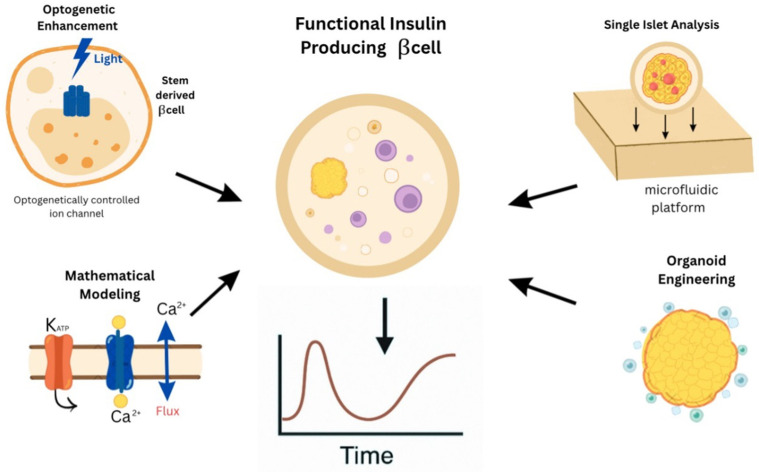
This figure summarizes key strategies for engineering functional β-cells: optogenetics enhances insulin secretion, single-islet analysis reveals functional variability, and organoid engineering improves transplantation outcomes. Mathematical modeling supports the understanding of calcium signaling. Together, these tools enable advanced diabetes cell therapies.

Advancements have been made, but obtaining functional equivalence between stem cell-derived β-cells and their native counterparts remains difficult. Many differentiation techniques produce insulin-secreting cells, although with lesser effectiveness and incomplete maturation than native β-cells [60]. Furthermore, difficulties such as immunological rejection, poor vascular integration, and limited long-term survival of transplanted cells continue to impede clinical development. To overcome these limitations, encapsulation techniques and advanced tissue engineering procedures are being investigated. Importantly, techniques that aim to imitate natural electrophysiological features, such as correct calcium flow and potassium channel activity, are expected to improve both GSIS and graft survival. Tools for enhancing engraftment through cellular scaffolding and co-culture systems add a layer of sophistication that may ultimately lead to a functional cure for DM [58,59]. As stem cell technologies mature and regulatory frameworks adapt, it is likely that a combination of these approaches will yield the most effective, safe, and scalable therapeutic solutions for insulin-dependent diabetes (Figure 4). The transition from stochastic self-aggregation to deterministic bioengineering represents a pivotal advancement in achieving functional β-cell maturation. While traditional 3D clusters often suffer from sluggish glucose-sensing kinetics and limited durability, the integration of microfluidic “organ-on-a-chip” platforms and 3D bioprinting allows for the precise mimicry of the high-perfusion pancreatic niche. These engineering approaches enhance GSIS by providing continuous nutrient exchange and hemodynamic shear stress, which act as mechanical cues for the electrophysiological maturation of voltage-gated calcium channels and K_ATP signaling. Furthermore, the strategic move toward tissue-specific decellularized ECM bioinks, as opposed to generic synthetic hydrogels, reestablishes essential integrin-mediated signaling pathways, which directly govern the docking and exocytosis of insulin granules. By combining these scaffold-based strategies with vascular and mesenchymal co-cultures, researchers can bypass the diffusion limits that traditionally cause central necrosis, ultimately generating insulin-producing units that more faithfully mirror the biphasic secretion profile and metabolic robustness of primary human islets [45].

### 2.5. Why Are Adult/Tissue-Derived Pancreatic Organoids Not Yet a Mainstream Source of β-Cells?

Despite clear advantages like long-term expansion with genomic stability and favorable safety profiles, adult/tissue-derived pancreatic organoids are not yet a routine source of functional β-cells for therapy. Key barriers include limited and inconsistent input tissue, which constrains scale and lot-to-lot consistency even under standardized, GMP-compliant workflows [61]; lineage bias and heterogeneity across resident ductal/islet/Procr^+^ compartments that yield variable endocrine competence; and incomplete endocrine specification and maturation, where forced programs (e.g., NGN3 mRNA) can induce insulin expression but often with immature phenotypes relative to adult islets [21,22]. Collectively, these factors limit the reproducibility of glucose-responsive function, helping explain why adult/tissue-derived organoids remain more mature as platforms for modeling and discovery than as a primary, scalable source of transplant-grade β-cells, despite their strong expansion and stability attributes [4].

**Table 1:** (**A**) Adult/tissue-derived pancreatic organoids: derivation, expansion, and functional readouts. (**B**) hPSC-derived β-like cells/SC-islets: formats, characterization, and functional readouts.Abbreviations: NR, not reported; GSIS, glucose-stimulated insulin secretion; MSCs, mesenchymal stromal cells.

(A)								
Starting Source/Approach	Selection/Enrichment	Matrix & Media (Examples)	Passage Range & Clonality	Genomic Stability	Functional/Maturity Readouts (Incl. GSIS)	Strengths	Limitations	References
Adult ductal-derived human pancreas organoids (hPOs)	Ductal enrichment (e.g., KRT19/SOX9); manual pick vs. filtration	Matrigel; EGF/Noggin/RSPO	Long-term passages (≥P10); clonal lines achievable	Reported stable by WGS/karyotype over passages	Endocrine conversion potential is context-dependent; GSIS: NR in expansion state	Robust long-term expansion; donor cryo-recovery; clonal workflows	Endocrine yield/maturity variable without additional induction	[4]
Islet-resident Procr^+^ progenitor organoids	PROCR (CD201)^+^ sorting	Matrigel; organoid medium	Long-term expansion; high clonogenicity	Reported stable during culture	Maintains endocrine competence; GSIS: NR (reported insulin production; fold not specified)	Defined resident progenitor; scalable lines	Requires cell sorting; translational protocols are still maturing	[22,23]
Dissociated human islet cells re-aggregation (pseudoislets) ± MSCs (hanging-drop)	Size control by input cell number; no marker selection	Hanging-drop (scaffold-free); co-culture with MSCs	No passaging; size-controlled clusters	NR	In vivo insulin secretion shown; GSIS: fold NR; size affects viability	Uniform size; improved function/vascularization vs. random aggregates	Labor-intensive; scale-up challenges	[36]
Self-condensation of tissue fragments with endothelial/mesenchymal support	Tissue-fragment self-organization; pro-vascular co-assembly	Self-condensation culture; pro-angiogenic cues	NR for pancreas-specific passaging	NR	Improved vascularization of constructs; GSIS: NR	Enhances engraftment potential via vascularization	Originally shown across tissues, pancreas-specific metrics are limited	[31]
Islet-laden silk/other engineered matrices	None	Silk matrices; “soft” hydrogels	NR	NR	Enhanced cluster formation; GSIS: NR or study-specific	Tunable mechanics; improved survival	Protocol heterogeneity; variable endocrine readouts	[32]
GMP-compliant adult pancreas organoids	Ductal/progenitor	Xeno-free/GMP media & process	Scalable production	Process controls documented	Functional assays vary by lab; GSIS: NR	Translation-ready workflows	Still, limited endocrine maturation in many settings	[61]
(**B**)								
**Platform/Format**	**Cell Line(s)/Stage Notes**	**Enrichment/Clustering**	**Key Markers & Characterization**	**GSIS (In Vitro Unless Noted)**	**Maturity Features**	**Strengths**	**Limitations/Notes**	**References**
Directed differentiation (2D → transplant)	hESC/hIPSC pancreatic endoderm → β-like post-transplant	None	Stage markers (PDX1, NKX6.1, etc.)	Often robust in vivo glycemic rescue; in vitro fold varies/NR	Post-transplant maturation	First clinical-grade exemplars	Immature in vitro; transplant-dependent	[62]
Dynamic function protocol (SC-islets)	Multi-line protocols improving Ca^2+^/exocytosis	Re-clustering to islet-like size	Acquisition of dynamic responses	Improved vs earlier gen; exact fold varies/NR	Emerging biphasic features in subsets	Better kinetics & coupling	Line-to-line variability; incomplete uniformity	[12]
Multi-omics maturation (SC-islets)	Systems profiling of maturation trajectory	Size-controlled clusters	Transcriptome/proteome/metabolism	Fold varies/NR	Closer to adult features in subsets	Deep characterization	Residual immaturity; heterogeneity persists	[63]
Metabolic maturation axis	ERRγ pathway modulation	—	Metabolic benchmarks	Fold varies/NR	Improves oxidative metabolism	Mechanistic clarity	Protocol integration needed	[64]
Maturity benchmark	—	—	UCN3/MAFA as late markers	—	Marker framework for “mature” β	Clear readout	Marker ≠ full function	[65]
Bioreactor suspension (scale-up)	Suspension SC-islet production	—	Batch metrics reported	Fold varies/NR	Process control	Scale and consistency	Function varies by run	[66]

## 3. iPSC-Derived Pancreatic β-Cells

hESCs and iPSCs can be cultured as embryoid bodies in suspension systems, such as spinner flask bioreactors. Subsequent culture in low-attachment conditions facilitates the aggregation of hPSC-derived pancreatic and endocrine progenitors into 3D islet-like structures. The potential for generating insulin-producing β-like cells from patient-specific iPSCs is substantial, offering not only a viable source for autologous transplantation but also a robust platform for disease modeling, drug discovery, and mechanistic investigations [67]. Despite significant advancements, the field encounters persistent challenges regarding the functional maturation and clinical scalability of these cells [68]. Recent reviews emphasize that while iPSC technology has revolutionized disease modeling, achieving consistent and mature functional phenotypes remains a primary obstacle for treating diabetes and associated complex pathologies. These platforms encompass diverse physiological systems—including pancreatic, vascular, retinal, and renal models—to elucidate disease mechanisms and enable personalized screening for therapeutics such as GLP-1 receptor agonists and tirzepatide. Such progress is essential for advancing cell-replacement therapies for diabetes and its microvascular complications [69].

### 3.1. Pluripotent Stem Cell-Based Differentiation

Because they can replicate indefinitely and differentiate into almost any type of cell in the body, hPSCs are the gold standard for making islet lineage cells [70]. Breakthroughs in stem cell tech have enabled production of insulin-producing β-cells from both ESCs and iPSCs through a process called directed differentiation [71]. For instance, researchers can take embryonic stem cells and use them to make pancreatic endoderm cells, which express genes like PDX1, FOXA2, HNF6, and NKX6.1. These cells then go on to make fully functioning endocrine β-cells after they have been transplanted into mice [72].

### 3.2. Stepwise Differentiation: Recapitulating Developmental Pathways

The development of insulin-producing pancreatic β-like cells involves emulating the pancreas’s development from an embryo. Most of the differentiation protocols are structured in a way to replicate the original sequence of development. This means cell development progresses from the very beginning: pluripotent cells go on to become definitive endoderm (DE), then foregut endoderm, then pancreatic progenitors, endocrine progenitors, and eventually the final stage of β-like cells [73,74]. The very first stage in this process is to get the cells to turn into DE by using high concentrations of Activin A, Wnt3a, or a chemical that mimics it called CHIR99021. This is all done in a low-serum or no-serum environment. The expression of markers such as SOX17, FOXA2, and CXCR4 confirms successful DE induction [62]. Afterwards, cells are treated with retinoic acid (RA), FGF10, and inhibitors of BMP (Noggin) and Hedgehog signaling (e.g., KAAD-cyclopamine), which guide differentiation into a posterior foregut identity marked by expression of HNF1β, HNF4α, and PDX1 [75].

This pancreatic identity is further solidified with expression of PDX1 and NKX6.1, which define a multipotent progenitor population [76]. Endocrine specification begins with activation of NEUROG3 (NGN3), a key regulator of endocrine lineage commitment [77]. Additional transcription factors such as NEUROD1, ISL1, and PAX4 are known from developmental studies to guide subsequent beta-cell differentiation [78]. Finally, beta-like cells begin to express insulin (INS), C-peptide, MAFA, GLUT2, and PCSK1, though the extent of functional maturation remains a critical limitation [5,68].

### 3.3. Maturation Deficits: The Challenge of Functional Competence

A persistent challenge is the limited functionality of derived β-like cells. In vitro, these cells often secrete insulin at basal levels without appropriate glucose responsiveness, and commonly co-express multiple pancreatic hormones such as glucagon and somatostatin, which are features more characteristic of fetal β-cells [79,80]. Comparative transcriptional studies show that insulin-producing pancreatic β-cells tend to have lower levels of genes linked to insulin release, glucose metabolism, and oxidative phosphorylation when compared against adult islets [63]. Notably, key markers of cell maturity, such as MAFA, UCN3, and estrogen-related receptor gamma (ERRγ), are usually under-expressed when grown in the lab [64]. ERRγ has turned out to be a key regulator in getting beta cells to program metabolically. In addition, its role in mitochondrial biogenesis and enhancing GSIS is pretty well understood, and forcing ERRγ into cells makes them function better [64,65,81]. Furthermore, the fact that these cells are struggling to get a good flow of calcium into the cell further highlights how immature they are. Calcium influx is essential for insulin vesicle fusion and release, yet IPCSCs often show delayed or blunted calcium responses upon glucose stimulation [82,83].

### 3.4. In Vivo Maturation and Implantation Strategies

Transplantation of pancreatic β-cells that originated from iPSCs into immunodeficient mice has shown an impressive record of enhanced maturation and functional performance. The location where they get implanted is a major factor in engraftment success [60,84]. Within weeks of engraftment, these cells exhibit increased insulin content, upregulation of key beta-cell genes, and restoration of normoglycemia in diabetic mice [12].

The renal subcapsular space is the most widely used site due to its rich vascularization and accessibility, making it particularly good for releasing a lot of insulin and rapidly getting the diabetes under control in these models. There are studies showing that these β-cells mature properly and can respond to sugar levels after being implanted under the kidney capsule [5,13,72,84]. The epididymal fat pad, another commonly used site in male mice, also provides a vascularized adipose niche conducive to graft survival and was utilized successfully by Kroon et al. and Schulz et al. [85,86]. In contrast, the subcutaneous space, though less vascularized by default, becomes favorable when combined with encapsulation devices or prevascularization techniques. These strategies facilitate oxygen and nutrient exchange while shielding the graft from immune attack, as demonstrated by Rezania et al. and Bruin et al., who reported functional insulin secretion and normoglycemia post-transplantation [72,87]. Collectively, these sites provide diverse environments that can be leveraged to optimize the maturation and therapeutic efficacy of iPSC-derived beta cells in vivo (Table 2).

### 3.5. Genomic and Epigenetic Considerations

Differentiation efficiency and cell fate stability are influenced by both the somatic origin of the donor cells and the epigenetic memory retained after reprogramming [88]. This leftover memory, a kind of legacy from the cell’s original home as a somatic cell, including the way those cells were expressing genes and had their DNA modified, can actually steer the direction of the reprogrammed cells’ development, or push them towards becoming one type of cell over another. For example, iPSCs derived from pancreatic tissues often demonstrate a lineage bias that facilitates redifferentiation into beta cells [89].

The genetic engineering tools based on CRISPR have become a valuable tool for enhancing the function and differentiation of pancreatic β-like cell stem cells. By enabling the editing of genes in these cells with precision, technologies like CRISPR-Cas9 and CRISPR-Cpf1 have been used to find the key players involved in how the pancreas develops, how long the cells that make insulin survive, and how well they can respond to glucose [90]. For instance, Wei et al. used a genome-wide CRISPR knockout screen to identify the vitamin D receptor (VDR) as a crucial factor for β-cell maintenance and function. Their study revealed that vitamin D can switch BAF chromatin remodeling complexes, protecting β cells both in vitro and in vivo [91]. Similarly, Li et al. conducted a genome-scale CRISPR screen and discovered that JNK–JUN signaling acts as a barrier to endoderm differentiation from pluripotent stem cells, suggesting that pathway inhibition may improve differentiation efficiency [92]. Zhu et al. demonstrated that editing key lineage determinants such as PDX1, NGN3, and RFX6 in hPSCs allowed dissection of their roles in human, as opposed to murine, pancreatic development—highlighting, for example, the haploinsufficient role of PDX1 and the regulatory role of RFX6 in endocrine progenitor specification [93]. These genome-editing studies not only enhance our understanding of human pancreatic development but also provide a strategy to engineer more functionally mature and stable β-like cells for disease modeling and potential therapies.

### 3.6. Pancreatic Organoid Transplantation Clinical Trials

The clinical translation of pancreatic organoids and stem cell-derived β-cells has really accelerated in recent years, with 2024–2025 reviews capturing the state of the art, from encapsulated progenitor cell trials to those first-ever human trials of autologous iPSC-derived islets that are achieving insulin independence [94,95]. Early phase clinical trials have focused on safety, engraftment, and metabolic function of implanted organoids, with several key studies showing proof of concept. The VC-01™ trial (NCT02239354), which encapsulated pancreatic progenitor cells, was terminated due to poor engraftment. Early-stage devices have a hard time with vascular integration [96]. The VC-02™ study (NCT03163511) showed some C-peptide production in a few participants, but procedural complications and limited durability showed that better delivery systems were needed [97].

VX-880, a stem cell-derived islet therapy developed by Vertex Pharmaceuticals, is looking promising with insulin independence in a subset of Phase 1/2 participants [98]. VX-264 and VCTX211 (CRISPR edited, immune evasive organoids in a retrievable device) are next-generation approaches to eliminate immunosuppression and improve graft survival [99,100].

The Sernova Cell Pouch™ trial (NCT03513939) showed that pre-vascularized device niches can support islet viability, but long-term functional outcomes are still unknown [101]. Donislecel (NCT03791567), an FDA-approved allogeneic islet therapy, has proven the concept of cell replacement therapy in brittle T1D, but with ongoing immunosuppression [102]. In summary, there are three main obstacles faced: (1) mature organoids, (2) no chronic immunosuppression, and (3) scalable engraftment. A 2025 thorough review makes it clear that linking β-cell technology with the latest biomaterial engineering is crucial to get around these translation hurdles and move on to developing next-generation bioartificial pancreas systems [103]. Next steps are gene-edited organoids, biomaterials for vascularization, and combined immunomodulation to get to a functional cure.

## 4. Comparative Analysis and Tissue Sourcing

### 4.1. Sourcing Strategies and Biopsy Morbidity for Pancreatic Organoids

A systematic analysis of pancreatic organoid creation reveals that the primary bottleneck is the acquisition of viable adult tissue. Traditional pancreatic tissue biopsies carry a substantial risk of morbidity, including pancreatitis, hemorrhage, and leakage [104,105]. To circumvent these risks, alternative non-invasive sourcing methods are being developed. For instance, secretin-stimulated pancreatic juice collected from the duodenal lumen during endoscopy allows for the isolation of ductal epithelial cells to generate organoids without puncturing the organ [106]. While pancreatic juice-derived organoids offer a safe liquid-biopsy approach, they are primarily epithelial and lack endocrine density. Alternatively, following a total pancreatectomy, biopsies can be taken safely from the surgical material without risk to the patient, representing an ideal, high-yield mode of tissue-derived organoid creation [107]. These surgical and fluid-based alternatives provide patient-specific platforms without the risks associated with percutaneous needle punctures.

### 4.2. Structural vs. Scalable Advantages: Organoids vs. iPSC-Derived β-Cells

When comparing adult tissue-derived organoids to iPSC-derived platforms, distinct advantages and design principles emerge. Tissue-derived pancreatic organoids excel in architectural mimicry by preserving ductal and acinar microenvironments [4,108]. This structural integrity allows for robust modeling of exocrine diseases like cystic fibrosis and facilitates spontaneous host vascularization upon transplantation [109]. Furthermore, adult organoids offer a higher safety profile regarding genomic stability and lower tumorigenic risks compared to pluripotent models [4]. In contrast, iPSC-derived platforms offer unparalleled scalability and flexibility. While adult organoids are limited by scarce tissue availability and inconsistent endocrine yield, iPSCs provide an infinite starting source. Using bioreactor-based suspension systems, iPSC platforms can achieve massive cost reductions and generate billions of glucose-responsive islet-like clusters [66,110,111]. Moreover, patient-specific iPSC reprogramming enables autologous therapies and the modeling of developmental disorders, such as pancreatic agenesis [112]. However, residual undifferentiated pluripotent cells pose a continuous risk of teratoma formation, necessitating physical containment via macro-encapsulation or strict purification protocols [89,113,114]. Despite their different origins, both platforms share core engineering design principles. Both systems rely heavily on biomaterial-enhanced niches—such as decellularized pancreatic ECM, synthetic PEG hydrogels, and gelatin microwell chips—to drive maturation [108,115,116,117]. Moving forward, the field is witnessing a convergence where synthetic biology and advanced bioprinting are applied to both modalities. Trilineage differentiation protocols are being designed to push iPSC clusters toward the architectural complexity of organoids, while tissue-trapping print systems are giving adult organoids the scalability and vascular precision historically reserved for stem-cell engineering [118]. A detailed comparison between pancreatic organoids and iPSC-derived platforms is summarized in Table 3.

## 5. Challenges and Future Directions

**Figure 5:**
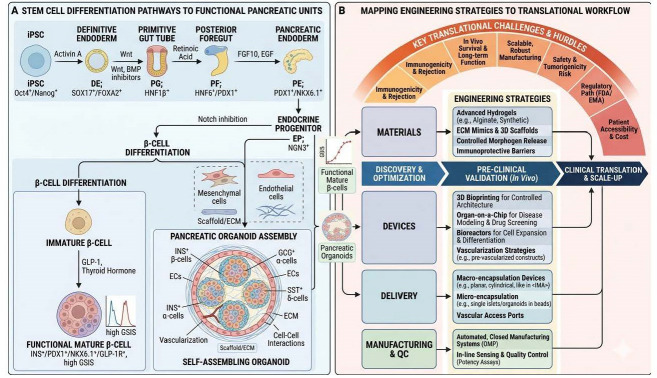
Part (**A**) delineates the intricate biological journey from pluripotent stem cells to functional insulin-producing units by mimicking the sequential signaling cues of embryonic development. This process begins with the induction of definitive endoderm and progresses through specific stages like the primitive gut tube and posterior foregut to reach the pancreatic endoderm marked by PDX1^+^ and NKX6.1^+^ expression. The schematic further illustrates a critical divergence where cells are either matured into isolated functional β-cells capable of high glucose-stimulated insulin secretion or assembled into complex 3D pancreatic organoids that incorporate mesenchymal and endothelial cells to better replicate the vascularized microenvironment of a native islet. Part (**B**) maps the diverse engineering strategies required to navigate the translational workflow and overcome significant clinical hurdles such as immunogenicity, scalable manufacturing, and regulatory approval. By integrating advanced biomaterials like immunoprotective hydrogels with sophisticated hardware like 3D bioprinters and large-scale bioreactors, researchers can bridge the gap between initial discovery and human application. This roadmap emphasizes the transition toward automated, closed-system manufacturing and in-line quality control sensors, ensuring that the lab-grown pancreatic units remain safe, potent, and accessible as they move through pre-clinical validation toward final clinical translation.

Despite significant progress in islet organoid development, several critical issues must be addressed before clinical implementation is feasible. These challenges can be categorized into four primary domains: vascularization, immune protection, functional maturation, and scalable manufacturing. Figure 5 illustrates the approach required to move from basic stem cell biology to a clinical therapy for diabetes.

### 5.1. Vascularization and Nutrient Delivery

Transplanted organoids lack an endogenous vascular network, which leads to hypoxia, nutrient deficiencies, and reduced long-term survival [94]. Without immediate perfusion, the core of the organoid often undergoes necrosis. To address this, bioengineering techniques using vascularized scaffolds and microfluidic devices aim to mimic the natural islet microenvironment and improve nutrient delivery [122,123]. Current engineering approaches include the co-culture of β-cells with endothelial cells to promote self-assembling capillary-like structures and the use of 3D bioprinting to create pre-defined vascular channels within the graft.

### 5.2. Immune Protection and Graft Survival

Allogenic transplantation and the recurrence of autoimmunity in T1D remain major barriers [124,125,126,127,128,129,130,131]. Beyond adaptive T-cell attacks, lessons from clinical islet transplantation highlight the Instant Blood-Mediated Inflammatory Reaction (IBMIR). This is driven by the expression of Tissue Factor (TF) on -cells, which triggers a rapid coagulation cascade upon contact with blood, leading to early graft deterioration [104,105]. Engineering strategies to combat these issues include advanced encapsulation devices and biomaterial scaffolds designed for localized immunomodulation. Furthermore, because iPSCs are uniquely amenable to complex genetic manipulation, researchers are using CRISPR/Cas9 to create cloaked cells by deleting HLA class I molecules or knocking out the TF F3 gene to prevent IBMIR [132].

### 5.3. Functional Maturation and Architectural Fidelity

Compared to native islets, current organoids often exhibit functional deficits, such as delayed functional onset after transplantation, abnormal glucose-stimulated insulin secretion kinetics, and temporary therapeutic effects [12,128,133]. Replicating the complex 3D architecture of the islet is crucial, as the endocrine niche requires specific ECM support and neuronal signaling for proper maturation [134]. Additionally, a major technical hurdle is the heavy reliance on Matrigel, a basement membrane extract derived from mouse Engelbreth-Holm-Swarm tumors. While Matrigel provides a rich suite of growth factors, its xenogeneic origin poses significant regulatory risks for human transplantation, and its inherent batch-to-batch variability hampers reproducibility [33,135]. Furthermore, Matrigel often fails to replicate the specific mechanical stiffness of the human pancreatic niche. To overcome this, researchers are developing defined Matrigel substitutes, including synthetic hydrogels (e.g., PEG, alginate) and recombinant protein-based gels that offer tunable biochemical and mechanical properties [108]. Notably, collagen matrices derived from fetal organs are emerging as a superior bio-instructive alternative. Unlike adult collagen, fetal-derived ECM is uniquely enriched with developmental signaling molecules and specialized glycosaminoglycans that more closely mimic the soft embryonic environment required for the transition of iPSCs into functional, glucose-responsive β-cells. These fetal-derived scaffolds provide the necessary architectural fidelity to improve the kinetic response of insulin secretion, which is often sluggish in current organoid models [79].

### 5.4. Scalable Manufacturing and Quality Control

Clinical translation requires universal islet products that can be produced at a scale of billions of cells per patient [136]. However, variability in growth factor concentrations and lab-to-lab protocols makes reproducibility difficult. The field is moving toward automated, closed-system manufacturing in stirred-tank bioreactors to ensure consistency. The field is moving toward automated, closed-system manufacturing in stirred-tank bioreactors to ensure consistency.

Overcoming current limitations will depend on the synergy between technological advancements in 3D culture systems and genetic engineering. As the pace of this field accelerates, the integration of modular bioengineering platforms will likely overcome these current limitations within the next few years, establishing islet organoids as a reliable source for transplantation therapy.

### 5.5. Ethical and Regulatory Considerations

The transition from lab-scale manual protocols to automated manufacturing requires “universal” islet products that do not require immunosuppression. The use of fetal-derived materials, while biologically superior, introduces specific ethical and sourcing considerations that must be balanced against their high therapeutic potential. Additionally, non-invasive sensing technologies and suicide gene techniques are being integrated into the workflow to address safety concerns, specifically the risk of tumorigenicity from remaining undifferentiated cells [14,120,137,138,139].

## 6. Conclusions

The past decade has seen a paradigm shift in the fabrication of pancreatic β-cell organoids and iPSC-derived clusters, moving from simple 2D cultures to complex, bio-instructive 3D systems. As discussed, iPSC-derived cells offer unparalleled scalability and the potential for “off-the-shelf” universal grafts, while tissue-derived organoids provide superior architectural fidelity and a lower risk of tumorigenicity. The novelty of the current landscape lies in the convergence of these two fields through advanced bioengineering; the same tools used to vascularize iPSC-islets are now being applied to stabilize the ductal-endocrine niche of organoids.

Despite this progress, the “bench-to-bedside” transition remains contingent on resolving the four primary bottlenecks identified in this review: vascularization, immune protection (both adaptive and innate/IBMIR), functional maturation, and scalable manufacturing. We conclude that the path to a functional cure for diabetes depends on the synergy between genetic editing and material science. By standardizing these bioengineering protocols and implementing non-invasive sourcing methods like pancreatic juice isolation, the field is moving toward a future where patient-specific, engineered pancreatic units can provide a permanent, insulin-independent solution for diabetes.

## Figures and Tables

**Table 2:** Common Implantation Sites for iPSC-Derived Pancreatic Β-Cells and Their Reported Outcomes in Mouse Models.

Implantation Site	Advantages	References	Reported Outcomes
Renal Subcapsular Space	Highly vascularized; easy surgical access	[5,33,84]	Enhanced maturation, increased insulin secretion, and normoglycemia in diabetic mouse models
Epididymal Fat Pad	Vascularized adipose niche; conducive to long-term graft survival	[85,86]	Functional graft survival and effective glycemic control
Subcutaneous Space	Clinically accessible; benefits from encapsulation or prevascularization to improve vascular support	[72,87]	Immune isolation, improved nutrient access, and reversal of hyperglycemia with encapsulated grafts

**Table 3:** Comparison of Pancreatic Organoids and iPSC-Derived Platforms.

Aspect	Pancreatic Organoids	iPSC-Derived Platforms	References
**Architecture & Function**	Mimic ductal and acinar architecture; robust exocrine disease and cystic fibrosis modeling	Flexible endocrine differentiation (e.g., β-cells); effective for developmental disorders and cancer modeling	[4,90,108,109,112]
**Engraftment & Vascularization**	Spontaneous vascularization post-transplant improves engraftment	Require biomaterials and encapsulation for survival and immune protection	[111,113,119]
**Tumorigenicity**	Low risk; stable genome over long-term culture	Higher risk due to residual undifferentiated cells; needs containment strategies	[4,85,120]
**Scalability Challenges**	Limited adult tissue availability; variable β-cell differentiation efficiency	High scalability in bioreactors; ~88.8% cost reduction in mass production	[61,66,86]
**Biomaterial Enhancements**	ECM and engineered hydrogel scaffolds improve complexity and maturation	Gelatin-based scaffolds and microwell chips guide pancreatic lineage differentiation	[90,108,111,115,117,121]
**Personalization & Disease Modeling**	Accurate for exocrine pathologies and cystic fibrosis	Enable autologous therapy and modeling of rare pancreatic disorders	[4,90,108,109,118]
**Diabetes Therapy Potential**	Can integrate into the host with a native-like structure	Require encapsulation and scaffold-induced angiogenesis	[71,96,111]
**Manufacturing & Innovation**	Bioprocessing improvements target reproducibility and standardization	Bioreactors reduce cost and enable high-volume production	[61,66,86]
**Converging Technologies**	3D-printed vascular traps and trilineage (ductal, acinar, endocrine) organoids are emerging	Rho kinase inhibitor-free protocols increase scalability	[90,116,118]

## Data Availability

No new data were created or analyzed in this study.
